# Water insecurity and gendered risk for depression in rural Uganda: a hotspot analysis

**DOI:** 10.1186/s12889-018-6043-z

**Published:** 2018-09-26

**Authors:** Christine E Cooper-Vince, Hawk Arachy, Bernard Kakuhikire, Dagmar Vořechovská, Rumbidzai C Mushavi, Charles Baguma, Amy Q McDonough, David R Bangsberg, Alexander C Tsai

**Affiliations:** 1 Massachusetts General Hospital, Boston, MA USA; 2 Harvard Medical School, Boston, USA; 3 Department of Environmental Management, Harvard University, Cambridge, USA; 4 Mbarara University Science and Technology, Mbarara, Uganda; 5 Johns Hopkins Bloomberg School of Public Health, Baltimore, USA; 6 Oregon Health Sciences University-Portland State University School of Public Health, Portland, USA

**Keywords:** Water insecurity, Depression, Geospatial, Gender, Sub-Saharan Africa

## Abstract

**Background:**

Water insecurity is linked to depression in low- and middle-income countries (LMICs), though it remains unclear how geospatial clustering of water insecurity in rural regions is associated with risk for depression.

**Methods:**

We conducted a population-based survey of a rural parish in southwestern Uganda (*N* = 1603) to evaluate the joint geospatial clustering of water insecurity and risk for depression among men and women living in rural Uganda.

**Results:**

Geospatial clustering of self-reported water insecurity and depressive symptoms was found to be present among both men and women. Depression hotspots were more often observed near water insecurity hotspots among women, relative to men. Multivariable regression revealed that residing in a water insecurity hotspot significantly increased risk for depressive symptoms among women, but not among men.

**Conclusions:**

Residing in a water insecurity hotspot is associated with greater risk for probable depression among women, but not among men, pointing to the need for focused depression screening among women residing in water insecure households.

## Background

Major depressive disorder is one of the leading cause of the disease burden globally and in sub-Saharan Africa measured in disability-adjusted life-years [1]. In general, the prevalence of depression is greater among women, an epidemiological finding that can potentially be explained by genetic and environmental risks and their interaction [2–4]. Women living in low and middle income countries (LMICs) are at increased risk for common mental disorders including depression, in part due to the high prevalence of poverty-related stressors that disproportionately affect women, such as food insecurity, limited education, financial hardship, intimate partner violence, and high fertility rates [5–11]. Furthermore, contextual effects, including living in a region without geographic proximity to health care and/or lacking access to daily necessities have been shown to contribute to depression [12–14]. Similar findings have been documented for other non-communicable diseases [15, 16]. Collectively, these findings suggest that women in rural settings within LMICs are at particularly high risk of poor physical and mental health outcomes.

Nearly 350 million people in sub-Saharan Africa lack reliable access to safe drinking water [17]. Water insecurity, defined as the limited or uncertain availability of safe water acquired via culturally acceptable means, has been associated with depression and anxiety among women in LMICs, as women bear the primary burden of ensuring household access to water for drinking and other household uses [18–26]. The distress related to water insecurity is in part explained by women’s increased difficulty fulfilling domestic roles (e.g., washing, cleaning, and cooking), maintaining hygiene, and providing hospitality [18], all of which are considered to be significant aspects of role functioning for East African women [27]. Accordingly, women residing in LMICs have reported greater concern about water access than their male counterparts [20, 24, 28].

Water insecurity, like food insecurity, is typically measured at the household level [24, 28–30] and is unlikely to affect individual households in isolation, given that distance from water source, elevation and accessibility are geospatially clustered [20, 24]. However, different households, and different individuals within a household, may have differential access to resilience resources that could buffer them against the deleterious effects of household-level exposures, so it remains unclear how an individual’s water security status relative to their neighbors’ is associated with risk for depression. A study of food insecurity in rural Uganda showed that instrumental support (e.g. provision of goods, labor, or money) from others within the social network was protective against food insecurity-related depression [8]. This finding could suggest that close proximity of water secure neighbors capable of offering such instrumental support (e.g., sharing water, lending money to pay for water fetching services) may protect water insecure individuals against risk for depression. However, such a hypothesis has yet to be evaluated empirically.

In the present study, we aimed to evaluate the joint geospatial clustering of water insecurity and depressive symptoms among men and women living in rural Uganda. We hypothesized that residence in an area with a high density of water insecure households would increase risk for depressed mood. Further, as women bear the primary responsibility for water acquisition and management, we hypothesized that this effect would be stronger for women relative to men.

## Method

### Study site

The study was conducted in a rural parish in Mbarara District, Uganda, located approximately 20 km outside of Mbarara Town, the district’s primary commercial hub and 260 km southwest of the capital city of Kampala. A parish is the second lowest administrative unit in the Ugandan government administrative structure, typically comprised of approximately 5–10 villages. The parish in which this study was conducted is comprised of 8 villages. The economy is based primarily on subsistence agriculture, and both food and water insecurity are common [24, 31]. The parish is contained within an approximately 6 km radius and has limited access to clean water, with a total of 89 water sources of varying quality distributed unevenly throughout the parish [24].

### Participants

We adopted a whole-population study design. All men and women were residents of a rural parish in Mbarara District, Uganda. Parish members were eligible to participate if they were over the age of 18 years (or were emancipated minors between 16 and 18 years of age), considered the Parish to be their primary place of residence, and were capable of providing consent for study participation. We excluded individuals younger than 18 years of age who were not emancipated minors; as well as those who could not communicate with research staff, e.g., due to deafness, mutism, or aphasia; psychosis, neurological damage, acute intoxication, or intellectual disabilities (determined in the field by non-clinical research staff in consultation with a supervisor). Potential participants meeting these eligibility criteria were approached by a research assistant fluent in the local language (Runyankore) to request their participation in the study. Written informed consent for study participation was obtained from those meeting eligibility criteria and expressing interest. Those who could not sign their names were permitted to indicate consent with a thumbprint. Once enrolled, each study participant completed an individual interview in a private location beyond the earshot of others. Surveys were completed from 2014 to 2015.

Ethical approval for all study procedures was obtained from the Partners Human Research Committee, Massachusetts General Hospital; and the Institutional Review Committee, Mbarara University of Science and Technology. Consistent with national guidelines, we received clearance for the study from the Uganda National Council for Science and Technology and from the Research Secretariat in the Office of the President.

### Measures

*Water Insecurity* (WI) was measured with the Household Water Insecurity Access Scale (HWIAS) [24]. The HWIAS is a self-report 8-item measure of household water insecurity, with possible total scores of 0–24. The HWIAS was developed based on the items of the Household Food Insecurity Access Scale [29], and was designed to elicit perceptions of insufficient quantity or quality of water, feelings of uncertainty or anxiety over water access, and strategies for coping with insufficient water for completing water-based tasks. The HWIAS was developed for use in the target population in southwestern Uganda, showing strong evidence of a coherent factor structure, reliability, and construct validity [24].

*Depression symptom severity* was assessed with the Hopkins Symptom Checklist-Depression Subscale (HSCL-D). The HSCL-D is a 15 item self-report of depressive symptoms derived from the 25-item short form of the HSCL that assesses both anxiety and depression [32]. The HSCL-D assesses the frequency of depressive symptoms in the past week. The version of the HSCL-D used in our study was modified for the local context; one item from the original scale (“feeling trapped”) was dropped due to poor performance in this context, and one item was added (“don’t care what happens to your health”; [33]). This version of the HSCL-D has shown strong evidence of reliability and validity in the local context in southwestern Uganda [8, 34–36]. The HSCL-D values were then used to classify study participants as being at risk for “probable depression” based on a threshold value of > 1.75, which is the threshold most conventionally applied in the literature [37].

*Self-reported overall health* was assessed using a self-report on a Likert scale in response to the question “How is your health in general?” Participants rated their health on a 4-point scale ranging from 1 = “Very good” to 4 = “Very bad”.

*Latitude, longitude, and elevation* coordinates, based on the World Geodetic System 84 standard [38], were obtained for each participant’s household, each identified water source, and major roadways within the parish.

### Analytic plan

#### GIS analysis

The residential locations of each participant who provided GPS coordinates (*N* = 696 households, 1603 participants) were visualized using ArcMap 10.3. The Getis–Ord General G global clustering statistic was used to identify significant spatial clustering of water insecurity and depression symptom severity [39, 40]:

$$ G\ (d)=\frac{\sum_{i=1}^N{\sum}_{j=1;j\ne i}^N{w}_{ij}{x}_i{x}_j}{\sum_{i=1}^N{\sum}_{j=1;j\ne i}^N{x}_i{x}_j} $$

where *G(d)* is the Getis-Ord General *G* value at critical distance (or neighborhood) *d*; *N* is the number of geographical areas; *i* and *j* are indices of geographical areas; *x*_*i*_ and *x*_*j*_ denote the variable values for areas *i* and *j*; and *wij* is the association weight for areas *i* and *j* at distance *d*.

Prior to using the depression data in the geographic information system (GIS) analysis, we fitted a multiple regression model to estimate a predicted depression symptom severity value for each participant adjusted for age, marital status, level of education, and household asset wealth. We estimated predicted values of water insecurity for each study participant using a multiple regression model adjusting for the same covariates. Additionally, because the HWIAS measures water insecurity at the household level, in households with multiple respondents, the mean household water insecurity scores for women and for men were substituted for individual level reports to minimize artificial inflation of clustering within households. We also assumed that observations taken at the same data collection location were considered “neighbors.”

To examine spatial clustering, a local version of the Getis–Ord statistic, the Gi* Statistic, was used. This statistic generates a value for each observation that represents the extent to which that observation is clustered with other observations that have similar values for that variable. A significance test analogous to the one described above (for the global clustering statistic) was applied to each observation. The advantage of the local test is that individual observations can be mapped, with the z-score values used as the map theme. This mapping allows visual inspection of the spatial clustering and interpretation of the results with regard to spatial distributions of other variables.

In this study, a range of neighborhood distances were tested because there is no known value of what constitutes a “neighborhood” in rural Uganda and because there are no secondary data from which to obtain neighborhood distance values. The parish represents 8 “villages” for administrative purposes, but in practice (i.e., as interpreted by residents) these administrative boundaries are often fluid.

#### Regression analyses

Based on the z-score derived from the GIS analysis, each participant was classified as either residing or not residing in a water insecurity hotspot. We fitted multivariable Poisson regression models with robust estimates of variance, following the “modified Poisson” approach as described by Zou [41] to estimate the association between probable depression and living in a water insecurity hotspot. When fitting models to data with a binary dependent variable specified, this approach allows for the exponentiated regression coefficients to be interpreted as risk ratios rather than as incidence rate ratios. Analyses were stratified by sex, given the theoretical motivation discussed in the introduction of this article. To formally test for effect modification by sex, we fitted the regression model to the pooled data but included a main effect for sex and a product term for the interaction between sex and living in water insecurity hotspot. Participant age, level of education, marital status, household asset wealth, and self-reported overall health were entered as covariates.

To assess the robustness of our findings to potential confounding by unobserved covariates, we applied the methods proposed by VanderWeele and Ding [42] to determine the E-value, defined as the minimum strength of association, on the risk ratio scale, that an unmeasured confounder would need to have with both probable depression and residence in a water insecurity hotspot to fully explain away the relationship between the two, conditional on the measured covariates. Additionally, to determine whether our findings were driven by selection of a culturally arbitrary threshold cutoff for the HSCL-D, we conducted a sensitivity analysis by specifying the HSCL-D score as a continuous dependent variable in a multivariable linear regression model. This model estimated the association between the degree of depression symptom severity and living in a water insecurity hotspot. Participant age, level of education, marital status, household asset wealth, and self-reported overall health were entered as covariates.

## Results

### Descriptive statistics

Of 1814 study participants in the population, latitude and longitude coordinates were obtained for 1603 participants. Of these, 56% were women, 77% were married or cohabiting with their primary partner, and 67% had not completed primary education. The mean depression symptom severity score was 1.72 (SD = 0.49) among women and 1.47 (SD = 0.37) among men. More than one-third (38%) of women, and 17% of men, had depression symptom scores above 1.75, indicating a probable depression diagnosis. The mean water insecurity score reported by women was 8.24 (SD = 6.93) while the mean WI reported by men was 7.35 (SD = 6.49).

### Geospatial clustering of depression and water insecurity

**Fig. 1:**
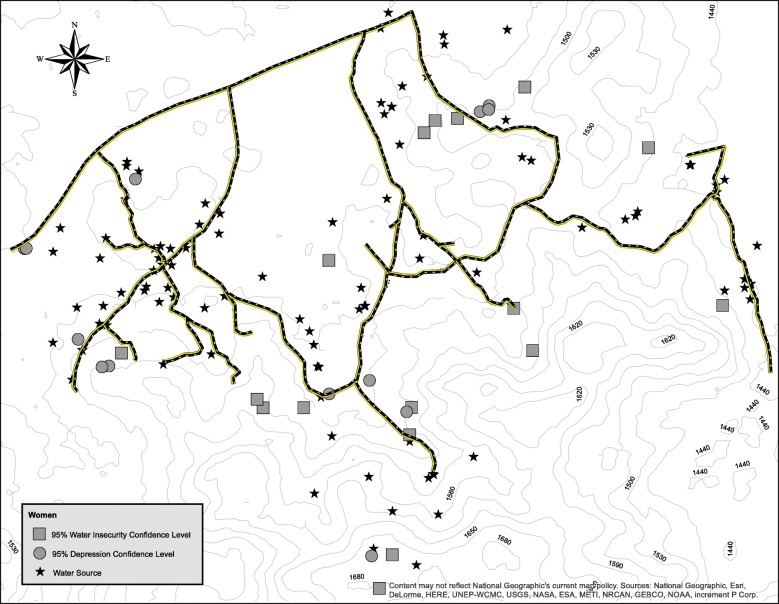
Geospatial clustering of depression and water insecurity among women

**Fig. 2:**
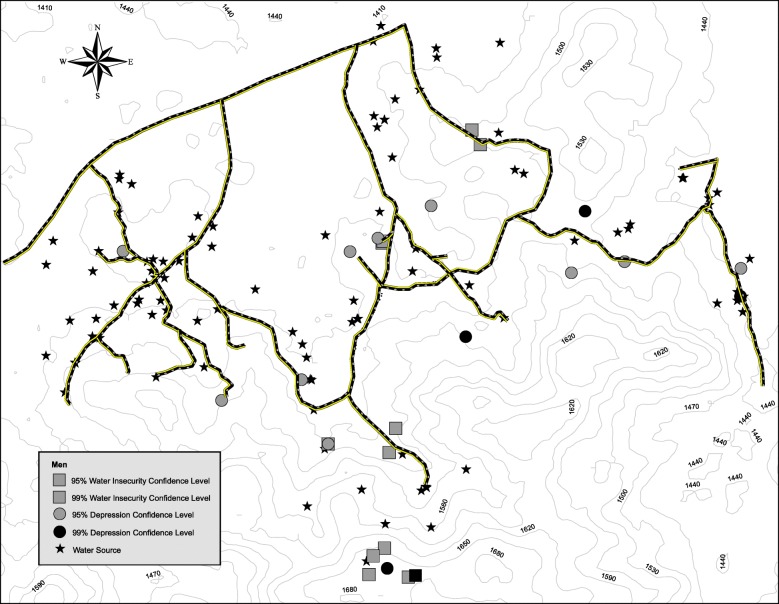
Geospatial clustering of depression and water insecurity among men

Maps showing the clustering of depression symptom severity and water insecurity are presented in Figs. 1 and 2. Significant clustering of water insecurity was observed among women (Z-score: 2.58, 54 m, *p* = .005) as well as men (Z-score: 2.61, 52 m, *p* = .005). Significant clustering of depression symptom severity was also observed among women (Z-score: 2.67, 60 m, *p* = .004) and men (Z-score: 2.60, 65 m, *p* = .005). Visual inspection revealed that clusters (“hotspots”) of elevated depression symptoms co-occurred with clusters of high water insecurity among women and men but co-occurred more frequently among women.

### Geospatial clustering of water insecurity and risk for probable depression

Multivariable Poisson regression analyses, stratified by sex, revealed that residence in a water insecurity hotspot is associated with increased risk of probable depression among women but not among men (Table 1). Specifically, women who reside in a water insecurity hotspot have 70% higher risk (adjusted relative risk ratio [ARR], 1.70; 95% CI 1.20, 2.40; *p* = .003) of probable depression, compared with women who do not reside in water insecurity hotspot. However, men who reside in a water insecurity hotspot are not at increased risk for probable depression (ARR 1.05; 95% CI 0.44, 2.47; *p* = .92). In a pooled regression model, we included a main effect for sex and tested for an interaction between sex and living in a water insecurity hotspot, but the estimated coefficient on the product term was not statistically significant (*p* = .08).

**Table 1:** Residing in a water insecurity hotspot as a predictor of probable depression

	Women			Men		
	IRR	95% CI		IRR	95% CI	
Living in WI hotspot	1.70	1.20	2.40	1.05	0.44	2.47
Age	1.00	0.99	1.00	1.00	0.99	1.01
Level of Education 1	1.15	0.91	1.45	0.78	0.47	1.30
2	0.86	0.64	1.17	0.60	0.33	1.08
3	0.87	0.62	1.21	0.64	0.35	1.16
Marital Status 1	1.00	0.82	1.23	1.37	0.89	2.10
2	0.64	0.45	0.92	0.70	0.43	1.14
Assets wealth	0.97	0.92	1.03	0.98	0.90	1.06
Overall health	0.53	0.45	0.62	0.46	0.34	0.63

To assess the extent to which our findings about water insecurity and probable depression among women could be explained by unmeasured confounding, we calculated the E-value. This analysis determined that only an unmeasured confounder that was strongly associated with probable depression and residing in a water insecurity hotspot among women, above and beyond the measured covariates, could explain away the coefficient (E-value = 2.79; 95% CI, 1.69–4.23). That is, the estimated risk ratio of 1.70 among women could only be explained away by an unmeasured confounder that was associated with both residing in a water insecurity hotspot and probable depression by a risk ratio of 2.79 fold each, above and beyond the measured confounders.

We obtained qualitatively similar findings in a sensitivity analysis conducted by fitting multivariable linear regression models to estimate the association between level of depression symptom severity and living in a water insecurity hotspot. Women who reside in a water insecurity hotspot were found to have higher depression symptom scores, relative to women who do not reside in a water insecurity hotspot (*b* = .26, 95% CI .03, .48, *p* = .024). This estimated coefficient was .26/1.72 = 15% of the sample mean among women, and .26/.49 = 0.53 standard deviation units, suggesting an association that is medium-sized in magnitude. Men who reside in a water insecurity hotspot did not experience increased depression symptom severity (*b* = .12, 95% CI -.09, .33; *p* = .26). When we fit the multivariable regression model to the pooled sample of men and women, the interaction between sex and living in a water insecurity hotspot was not statistically significant (*p* = .13).

## Discussion

In this cross-sectional, population-based study of women and men in rural Uganda, we found strong evidence of geospatial clustering of water insecurity and depression symptom severity among both men and women. Depressive symptom hotspots were more often observed near water insecurity hotspots among women, relative to men. In multivariable regression models adjusting for potential confounding by socioeconomic and health status, we found that residing in a water insecurity hotspot is associated with 70% greater risk for probable depression among women, but not among men. This finding was robust to a sensitivity analysis that did not define probable depression at the customary threshold score. Further, this relationship could only be explained away by an unmeasured confounder that was strongly associated with both residing in a water insecurity hotspot and probable depression, above and beyond the measured confounders.

Our findings about the association between water insecurity hotspots and probable depression are consistent with previous literature linking degraded land quality to increased depression and suicide among people residing in rural regions who rely on agricultural productivity for their livelihoods [43, 44]. Further, the fact that this estimate was statistically significant only among women is consistent with work identifying greater concern and distress related to limited and unreliable access to potable water among women, relative to men [18, 20, 24, 45] and can likely be explained by women’s primary role in water procurement and utilization for household tasks in sub-Saharan Africa, such as cooking, laundry and bathing children [21, 23]. Insecure access to potable water impedes women’s ability to complete their roles within the home and community, which in the local context significantly contributes to social shaming, worry and a negative self-concept. However, as men’s performance in culturally prescribed roles within the home and community is less closely tied to daily water-related chore completion, it would be expected that -- consistent with our data -- their emotional distress would not be associated with water insecurity.

These divergent findings by sex are potentially consistent with the well-established finding that social supports buffer against the development of depression [46, 47], particularly for women relative to men [48]. Further, previous work from the local context has shown that instrumental social support (i.e., the provision of money, materials goods, or services) is specifically what drives the buffering effects of social support against depression among resource insecure women [8]. Though there is a body of research that suggests that income inequality at the societal level increases risk for depression among economically-disadvantaged women, in resource-deprived settings, the ability and willingness of one’s proximal neighbors to provide even small amounts of instrumental support may play an important role in promoting health [49–51].

The present findings elucidate the associated mental health risks of resource deprivation in already low-resource communities. The local economy in Mbarara is driven principally by subsistence agriculture and animal husbandry, yet even in this setting there are hotspots of water insecurity. Women who reside in areas of the community that have the highest concentration of water insecurity are at greater risk for probable depression. This finding may be explained by women’s inability to procure instrumental support, particularly lending and receiving of water, from neighbors who are also themselves water insecure. Research from sub-Saharan Africa has shown that informal water sharing among neighbors is one method commonly utilized in the community to cope with water insecurity, further supporting this interpretation [52]. These findings suggest that interventions designed to increase secure water access in rural settings may benefit from facilitating informal water sharing methods between water insecure and water secure members of the community, as a means for coping with unexpected water shortages and pooled sharing of risks. Such strategies may be of particular benefit to depressed water insecure women, as they would enhance social support in a population already prone to social withdrawal and isolation.

Interpretation of our findings is subject to several limitations. First, depression symptom severity was assessed via self-report. Though more than one-third of women reported a depression score above the commonly accepted threshold for a probable depression diagnosis, these are known to overestimate the prevalence of major depressive disorder due to false positives [53, 54]. It is also possible that self-report of depression symptoms may bias against the detection of depression among men, relative to women, due to masking or externalization of depression symptoms associated with masculine norms in other settings [55–57]. Additionally, the study employed a cross sectional design, which limits our ability to infer a causal relationship between water insecurity and depression symptoms. Finally, we were unable to account for the effect of potential confounds such as family history of mental illness, domestic violence, and partner support on probable depression. However, the E-value sensitivity analysis suggests that our findings are unlikely to be fully explained by unobserved confounding given that only a variable with a strong association with both water insecurity and probable depression could completely explain away the estimated association.

These findings have important implications for policy. First, these findings point to the need for focused depression screening among women residing in water insecure households and locations with limited water access in rural Uganda. Such programs could leverage early childhood development home visiting programs and community health workers to identify households with poor water access and need for depression screening [58, 59]. Second, these findings bolster support for initiatives to increase access to potable water in rural Uganda, suggesting that enhancing reliable access to clean water is likely to benefit women’s mental health -- in addition to the commonly accepted motivations of improving physical health outcomes particularly among young children. Given the well-established risks for poor cognitive and behavioral outcomes among children conferred by maternal depression [60–62], increasing reliable access to potable water may also be an effective method for promoting healthy child development and reducing the burden on already highly-stressed healthcare and educational systems in low-resource settings.

## Conclusions

We found that residing in a water insecurity hotspot is associated with 70% greater risk for probable depression among women, but not among men, in rural southwestern Uganda. This finding was robust to a sensitivity analysis that did not define probable depression at the customary threshold score. These findings suggest the need for focused depression screening among women residing in water insecure households and suggest that increased access to potable water in rural Uganda is likely to benefit women’s mental health, in addition to physical health.

## Abbreviations

LMICs: Low and middle income countries
WI: Water insecurity
HWIAS: Household Water Insecurity Access Scale
HSCL-D: Hopkins Symptom Checklist-Depression Subscale
GIS: Geographic information system
ARR: Adjusted relative risk ratio
